# Correlations Between Oxygen Consumption, Ventilatory Mechanics, and Lung Ultrasound in Individuals with Post-COVID Syndrome

**DOI:** 10.3390/ijerph22121839

**Published:** 2025-12-09

**Authors:** Jéssica Gabriela Messias Oliveira, Samantha Gomes de Alegria, Isabelle da Nóbrega Ferreira, Iasmim Maria Pereira Pinto Fonseca, Matheus Mello da Silva, Beatriz Pereira dos Santos, Marcelo Ribeiro-Alves, Estêvão Rios Monteiro, Agnaldo José Lopes, Thiago Thomaz Mafort

**Affiliations:** 1 Post-Graduation Programme in Medical Sciences, School of Medical Sciences, Universidade do Estado do Rio de Janeiro (UERJ), Avenida Professor Manoel de Abreu, 444, 2º andar, Vila Isabel, Rio de Janeiro 20550-170, Brazil; jessica.messias@hupe.uerj.br (J.G.M.O.); samantha.alegria@hupe.uerj.br (S.G.d.A.); isabelle.nobrega@marinha.mil.br (I.d.N.F.); iasmimfonseca@souunisuam.com.br (I.M.P.P.F.); matheusmello@ufrj.br (M.M.d.S.); marcelo.ribeiro@ini.fiocruz.br (M.R.-A.); thiago.mafort@uerj.br (T.T.M.); 2 Rehabilitation Sciences Post-Graduation Programme, Centro Universitário Augusto Motta (UNISUAM), Rua Dona Isabel, 94, Bonsucesso, Rio de Janeiro 21032-060, Brazil; beatrizpereirasantos@souunisuam.com.br (B.P.d.S.); estevao.monteiro@ulife.com.br (E.R.M.)

**Keywords:** post-COVID syndrome, cardiopulmonary exercise testing, impulse oscillometry, spirometry, lung ultrasound

## Abstract

Introduction: Since COVID-19 primarily targets the respiratory system, it is essential to longitudinally monitor functional capacity and pulmonary function in individuals with post-COVID syndrome (PCS). This study aimed to evaluate the functional capacity of individuals with PCS during exercise using cardiopulmonary exercise testing (CPX) and examine its association with spirometry, impulse oscillometry (IOS), and lung ultrasound (LUS) parameters. Methods: Sixty individuals participated in this study. We assessed CPX, which measured peak oxygen consumption (VO_2_peak), in addition to IOS, spirometry, and LUS. Results: The mean VO_2_peak was 18.4 ± 5.9 mL/kg/min. Significant negative correlations were found between VO_2_peak and the following parameters: age (r_s_ = −0.465, *p* = 0.0002), body mass index (BMI, r_s_ = −0.354, *p* = 0.0056), resonance frequency using IOS (r_s_ = −0.312, *p* = 0.0193), and LUS aeration score (rs = −0.261, *p* = 0.0439). Conclusions: Patients with PCS undergoing CPX demonstrated impaired functional capacity. In these individuals, higher age and BMI were associated with lower VO_2_peak. Furthermore, alterations in LUS and IOS may also be linked to reduced VO_2_peak. These findings are promising, as they were obtained using a low-cost device. Further studies are needed to investigate the factors that influence oxygen consumption in PCS.

## 1. Introduction

Although it was first reported in 2019, many patients continue to experience post-COVID syndrome (PCS). PCS is characterised by persistent symptoms that last more than 12 weeks [1]. Indeed, between 35% and 90.5% of individuals who survive the initial infection continue to experience symptoms for weeks or months. The most frequently reported symptoms include chronic fatigue, dyspnea, chest pain, and neurocognitive disturbances, such as difficulty concentrating (“brain fog”), as well as sleep disturbances and depression [2]. Autonomic dysfunctions, such as tachycardia and postural orthostatic intolerance, have also been reported [3,4]. Despite the growing number of studies, significant gaps remain in our understanding of the impact of PCS on exercise capacity. Proposed mechanisms include peripheral muscle dysfunction, physical deconditioning, ventilatory impairment, and changes in ventilation dynamics; however, pathophysiological data are limited. The variability in how patients with PCS respond to exercise highlights the need for more integrated and personalised diagnostic approaches [5]. Identifying and monitoring these patients is essential to ensure appropriate treatment and mitigate the long-term functional impact. However, the lack of specific biomarkers, the symptom heterogeneity, and the interindividual variability complicate diagnosis, underscoring the importance of active and personalised clinical follow-up [6].

Since SARS-CoV-2 primarily targets the respiratory system [1,7], it is crucial to measure and monitor lung function longitudinally in individuals with PCS [8,9]. In clinical practice, spirometry is the most widely used pulmonary function test due to its simplicity and availability [8,9]. However, it is an effort-dependent test that requires active patient cooperation to ensure reliable results [10,11]. Recently, the impulse oscillometry system (IOS) has become more popular. IOS is a noninvasive, easy-to-use method capable of detecting small airway dysfunction (SAD), including in patients with PCS [12]. IOS uses small pressure variations superimposed on normal breathing, eliminating the need for forced expiratory manoeuvres. Evidence suggests that SAD, even with normal spirometry results, may be associated with ventilatory limitation during exertion in individuals with PCS [13]. Regarding imaging tests, lung ultrasound (LUS) is relevant in evaluating these patients because it poses a lower risk of contamination, does not expose subjects to radiation, and is inexpensive [14]. Since SARS-CoV-2 preferentially affects the peripheral areas of the lungs, which are accessible with LUS, this method has become an important tool in evaluating patients with PCS. Although few studies have been conducted on this population, LUS signals demonstrate high sensitivity and diagnostic accuracy, comparable to chest computed tomography (CT) [14].

One interesting way to evaluate individuals with PCS is by assessing their global functional performance, particularly through the cardiopulmonary exercise test (CPX), which is considered the gold standard for this purpose. In this population, a low ventilatory reserve is a relevant finding for many individuals with PCS [15]. Although studies have demonstrated reduced cardiopulmonary fitness (CPF) in individuals with PCS, the mechanisms underlying exercise intolerance remain unclear. These alterations may include physical deconditioning, dysfunctional hyperventilation, autonomic dysfunction, and residual pulmonary impairment. More specific investigations are required to understand the involved pathophysiology [16,17]. In this context, CPX is an essential tool for differential diagnosis of dyspnea and for directing evidence-based rehabilitation interventions, allowing for a more individualised approach [18,19]. Due to PCS’s multifactorial impact on CPF, accessible and reliable tools that assess oxygen consumption (VO_2_) during exercise are essential. Portable gas analysers are a valid alternative to conventional CPX, providing direct, real-time VO_2_ measurements and are increasingly being used. In people with PCS, this technology enables the early detection of ventilatory and metabolic changes, even during submaximal testing. This facilitates the identification of exercise intolerance and guides respiratory and functional rehabilitation strategies [16,20,21,22,23]. Portable gas analysers have standardised protocols that ensure reproducibility and safety during assessment; these are crucial factors for monitoring individuals with post-viral sequelae and persistent fatigue [24].

Importantly, portable gas analysers can be integrated with other well-established tools for assessing lung function, enabling a more comprehensive analysis of an individual’s overall function. Since VO_2_ measurements are not always available in clinical practice, it is essential to estimate them using simpler tools, such as pulmonary function tests and LUS. Several extrapulmonary manifestations of PCS can negatively impact VO_2_ measurements and, consequently, the CPF; these manifestations include lesions affecting the heart, blood vessels, skeletal muscle, and nervous system [2,3,4,5]. However, since the lungs are most affected during the acute phase of SARS-CoV-2 infection, we hypothesise that the CPF of individuals with PCS may be related to structural lung impairment and changes in resting lung function. Therefore, this study aimed to evaluate the functional capacity of individuals with PCS during exercise using CPX and examine its association with spirometry, IOS, and LUS parameters.

## 2. Materials and Methods

### 2.1. Study Design and Participants

From October 2021 to June 2024, a cross-sectional study was conducted on individuals with PCS who were over 18 years old and regularly followed at the Post-COVID Outpatient Clinic of the Piquet Carneiro University Polyclinic at the State University of Rio de Janeiro (UERJ) in Brazil. Patients presenting with continuous symptoms for more than three months after the acute phase of SARS-CoV-2 infection were included. The exclusion criteria included the following: absolute contraindications to CPX (e.g., acute myocardial infarction within the previous three to five days, unstable angina, uncontrolled arrhythmias, syncope, active endocarditis, acute myocarditis or pericarditis, severe aortic stenosis, decompensated heart failure, suspected aortic aneurysm, and arterial desaturation at rest on room air <85%) [25]; presence of lung diseases unrelated to PCS; smoking history exceeding 10 pack years; and inability to perform CPX. The study was approved by the Research Ethics Committee of the State University of Rio de Janeiro under the approval number CAAE-30135320.0.0000.5259, and all individuals signed a consent form.

### 2.2. Instruments and Measurements

The IOS measures resistance of respiratory system (Rrs) and reactance of respiratory system (Xrs) were made using an impulse oscillometer (Quark i2m, Cosmed, Rome, Italy). We evaluated the frequency range between 5 and 20 Hz. Since there is no consensus in the literature regarding the optimal resistive parameters for interpreting Rrs curves, the following Rrs parameters were used: Rrs at 5 Hz (R5), Rrs at 20 Hz (R20), and the heterogeneity of resistance between R5 and R20 (R5–R20) [26,27]. The R5 and R20 values were considered abnormal when ≥150% of the predicted value [28]. On IOS, lower frequencies (≤5 Hz) measure the viscous resistance of the airways, while higher frequencies (≥20 Hz) reflect the characteristics of the proximal airways [29]. Furthermore, we evaluated the resonance frequency (Fres), which is the frequency at which the inertial properties of the airways and the capacitance of the lung periphery are equal when the reactance is zero [30]. The normal range for Fres is 6–11 Hz [30]. During the IOS assessment, participants were instructed to sit with their head in a neutral position, with manual support on their cheeks, and with their nostrils occluded with a clip. Participants then performed spontaneous breathing for 40 s. The minimum acceptable coherence values are ≥0.9 Hz [11,26].

Spirometry was performed using a Vitatrace^®^ device (VT 130 SL, Codax LTDA, Rio de Janeiro, Brazil) according to the American Thoracic Society/European Respiratory Society standardisation guidelines and the manufacturer’s calibration guidelines [31]. The predicted values of forced vital capacity (FVC), forced expiratory volume in one second (FEV_1_), and forced expiratory flow during the middle half of the FVC manoeuvre (FEF_25–75%_) were calculated using the equations of Pereira et al. [32]. The results were expressed as percentages of the predicted values. Obstructive lung impairment was defined as an FEV_1_/FVC ratio of less than 70%, and restrictive lung impairment was inferred as an FVC of less than 80% of the predicted value in the absence of reduced expiratory flows [32].

LUS examinations were performed using a Mobile Trolley UMT-150 ultrasound device (Mindray, Shenzhen, China) equipped with a 3.5 to 5 MHz convex transducer operating in B mode. A 12-zone protocol was used to examine six regions in each hemithorax while participants were seated: two anterior, two lateral, and two posterior. During the evaluation, sonographic signs such as the presence of more than two B-lines, coalescent B-lines, and subpleural consolidations were observed. To quantify lung involvement, each of the six areas received a score based on the findings: one point was assigned for more than two B-lines, two points for coalescent B-lines, and three points for subpleural consolidations. The sum of the scores assigned to the twelve regions evaluated comprises the aeration score, which can range from 0 to 36 points [33].

The CPX was performed according to previous recommendations [34] using a cycle ergometer (Ergoselect 4 SN, Ergoline GmbH, Bitz, Germany) and the FitMate PRO™ (Cosmed, Rome, Italy) to measure VO_2_peak. [35] The modified Astrand protocol was adopted, in which participants cycled at 25 W at the beginning of the test. The work rate increased by 25 W every three minutes until the participant reached their maximum tolerance. The exercise stopped when the participant signalled marked dyspnea, muscle fatigue, or exhaustion [34]. Although the FitMate PRO™ does not have a carbon dioxide analyser, it increases the respiratory exchange ratio between 0.8 and 1.2 based on heart rate (HR) increases [36,37]. We used the VO_2_peak classification for CPF based on a sample of healthy Brazilian individuals, taking age and sex into account. For the present study, participants were classified into one of three categories: weak or very weak fitness, reasonable fitness, or good or excellent fitness [38]. The oxygen pulse (PuO_2_) was calculated by dividing VO_2_peak (mL/min) by HRpeak (beats/min) [39]. Low ventilatory reserve was defined as the difference between maximal voluntary ventilation (MVV) and minute ventilation (VE) at peak exercise of less than 15 L [39].

### 2.3. Statistical Analysis

A descriptive data analysis is presented in tables and expressed using appropriate measures of central tendency and dispersion. The Shapiro–Wilk test was used to verify normal distribution. Numerical data are expressed as the mean ± standard deviation or the median and interquartile range (IQR), while categorical data are expressed as the frequency (*n*) and the percentage (%). An inferential analysis using a Spearman’s correlation coefficient was performed to determine the association between CPF, resting lung function, and LUS aeration scores. The association between VO_2_peak and categorical variables was analysed using the Student’s *t*-test for independent samples. The significance level was set at 5%. Statistical analyses were performed using Jamovi statistical software, version 2.6.26 (The Jamovi Project, Sydney, Australia, 2024).

## 3. Results

Of the 63 participants with PCS who were eligible for the study, three were excluded for the following reasons: two due to orthopaedic alterations that prevented CPX, and one due to a smoking history greater than 10 pack years. Therefore, the sample consisted of 60 participants, 39 of whom were women (65%). The mean age was 50 ± 12.1 years. The mean body mass index (BMI) was 29.8 ± 5.3 kg/m^2^, with 9 (15%) being eutrophic, 25 (41.7%) being overweight, and 26 (43.3%) being obese. According to the World Health Organisation’s guidelines for categorising the severity of symptoms of the COVID-19 [40], 16 (26.7) patients experienced mild symptoms, 25 (41.7%) patients experienced moderate symptoms, 15 (25%) patients experienced severe symptoms, and four (6.7%) patients experienced critical symptoms. Fifteen (25%) individuals were hospitalised for COVID-19, and four (6.7%) required orotracheal intubation. For patients admitted to the hospital, the median length of stay was 16 (12–21) days. Twenty-five (41.7%) participants reported a history of physical activity, and 23 (38.3%) participants reported a history of respiratory physiotherapy. Regarding previous comorbidities, twenty-five (41.7%) and 15 (25%) participants reported hypertension and diabetes, respectively. Table 1 shows the sample’s anthropometric data, comorbidities, and other characteristics.

In IOS, the median R5 was 4.5 (3.8–6) cmH_2_O/L/s, the median R20 was 4.2 (3.4–5.3) cmH_2_O/L/s, and the median Fres was 17.5 (12.4–21.9) Hz. The median R5–R20 was 0.2 (−0.2–1) cmH_2_O/L/s. Twenty-four (40%) participants showed findings consistent with SAD. However, of the participants who underwent spirometry, 50 (83.3%) had normal results. Obstructive and restrictive impairments were observed in 8 (13.3%) and 2 (3.3%) participants, respectively. On the LUS evaluation, the median aeration score was 1 (0–5) point. On the LUS evaluation, mild impairment in the aeration score was the most frequent finding (*n* = 57, 95%), while only one (1.6%) participant had moderate alterations and two (3.3%) participants had severe alterations. Regarding CPX, all participants reached the exercise tolerance limit according to the established criteria. Total time and peak workload values were 7.5 (3.5–12.5) minutes and 75.6 ± 25.0 W, respectively. The mean VO_2_peak was 18.4 ± 5.9 mL/kg/min. Using the VO_2_peak classification for CPF [38], 58 (96.7%) participants were classified as having weak or very weak fitness, one (1.7%) as having reasonable fitness, and one (1.7%) as having good or excellent fitness. A low ventilatory reserve was observed in 26 (43.3%) participants. The average percentage of observed HR relative to expected HR was 82.8 ± 14.1%, considering the expected HR as 220 minus the age. Table 2 shows the data on lung function, ultrasound findings, and CPX.

Although the difference was not statistically significant, participants who required hospitalisation had lower VO_2_peak (15.8 ± 3.6 vs. 19.3 ± 6.3 mL/kg/min, *p* = 0.21). Those who required mechanical ventilation had lower VO_2_peak, and this difference was statistically significant (12.4 ± 2.9 vs. 20.8 ± 6.8 mL/kg/min, *p* = 0.015). Furthermore, participants with low ventilatory reserve had higher LUS aeration scores, and this difference was statistically significant (2 (1–7) vs. 0 (0–3) points, *p* = 0.026).

**Figure 1:**
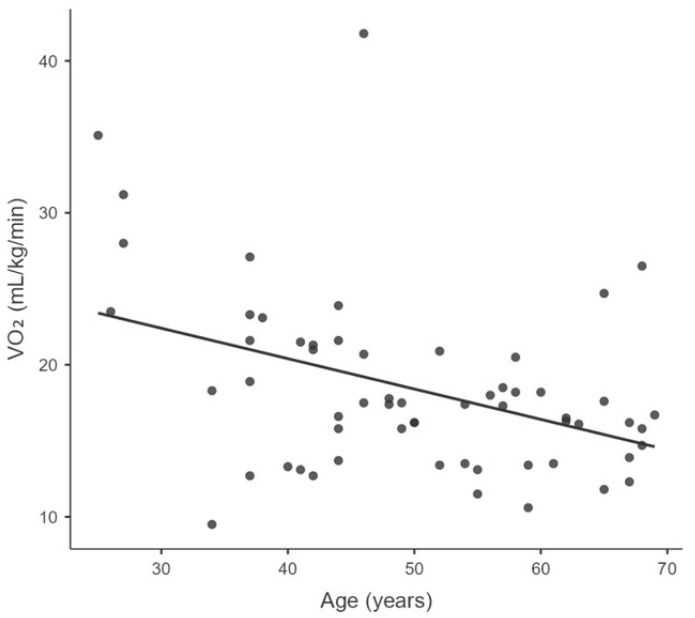
Correlation between the peak oxygen consumption (VO_2_peak) and age.

**Figure 2:**
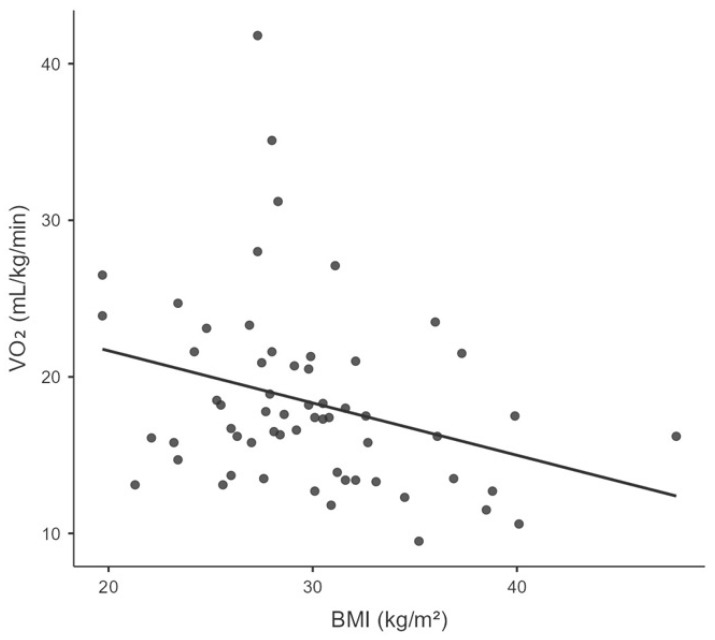
Correlation between the peak oxygen consumption (VO_2_peak) and body mass index (BMI).

**Figure 3:**
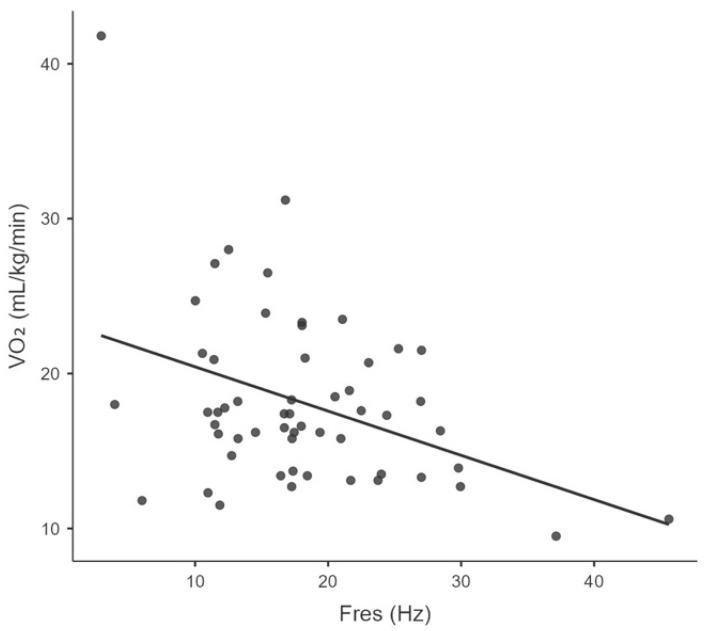
Correlation between the peak oxygen consumption (VO_2_peak) and resonant frequency (Fres).

**Figure 4:**
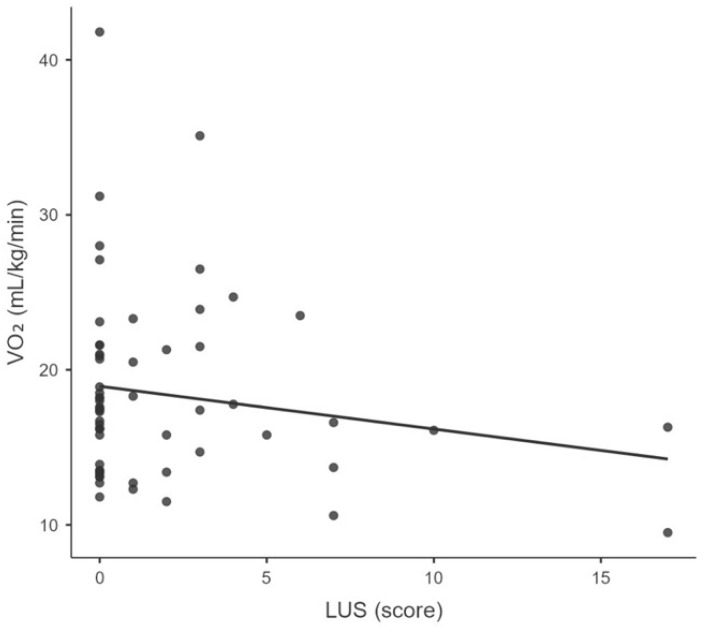
Correlation between the peak oxygen consumption (VO_2_peak) and lung ultrasound (LUS) score.

Significant negative correlations were found between VO_2_peak and the following parameters: age (r_s_ = −0.465, *p* = 0.0002), BMI (r_s_ = −0.354, *p* = 0.006), Fres (r_s_ = −0.312, *p* = 0.019), and LUS aeration score (rs = −0.261, *p* = 0.044). Table 3 and Figure 1, Figure 2, Figure 3 and Figure 4 illustrate the correlations between VO_2_peak, clinical characteristics, lung function, and LUS aeration score.

## 4. Discussion

Changes in both lung structure and mechanics are related to CPF in individuals with PCS. Alongside significant correlations between VO_2_peak and demographic and anthropometric data, our findings showed significant correlations between VO_2_peak, Fres, and LUS aeration score. To our knowledge, this is the first study to associate poor CPX performance (low VO_2_peak) with abnormalities in IOS and LUS in individuals with PCS.

The main data provided in the CPX are VO_2_ peak. This parameter is crucial for assessing cardiovascular health and is the gold standard for representing CPX. In our study, we obtained this result using the FitMate PRO™ and an accessible cycle ergometer. The mean VO_2_peak in this cohort (18.4 ± 5.9 mL/kg/min) was lower than normal values. Milani et al. [41] observed a median of 31.1 (23.6–37.5) mL/kg/min for sedentary adults. According to Herdy and Uhlendorf [42], the expected VO_2_peak values on a cycle ergometer for healthy, non-athlete adults in the Brazilian population are 39 ± 6.8 mL/kg/min for men and 30 ± 5.4 mL/kg/min for women. Braga et al. [43] compared individuals with PCS to a control group and found mean values of 30.9 and 31.1 mL/kg/min, respectively. Patients were stratified by disease severity in this study. Individuals with mild COVID-19 had a VO_2_peak of 35.7 mL/kg/min. This finding reinforces that the values obtained in our study were below the average for this population [44]. Milani et al. [45] found that exercise limitations in post-COVID patients predominantly resulted from peripheral muscle fatigue with pulmonary and cardiovascular contributions. This finding corroborates potential mechanisms related to reduced exercise tolerance, regardless of initial disease severity [45]. Physical deconditioning, myopathy, and chronotropic incompetence are among the contributing factors [43,44,45]. PCS is associated with subtle pulmonary sequelae, such as residual fibrosis, persistent inflammation, and small airway dysfunction. These sequelae can compromise gas exchange and ventilatory efficiency, thus reducing exercise tolerance [46]. In fact, over 40% of our sample exhibited low ventilatory reserve, suggesting that respiratory system inefficiency contributes to poor CPF in individuals with PCS.

Age is one factor influencing the CPF of individuals with PCS. In the present study, age was negatively correlated with VO_2_peak (r_s_ = −0.465; *p* = 0.0002) in CPX. Our results corroborate the findings of the systematic review with meta-analysis by Gomes-Neto et al. [47]. They found that, on average, VO_2_peak decreased by 0.20 mL/min/kg for each one-year increase in the mean age of the studies [46,47,48]. In other words, the older the individual, the poorer their functional capacity. These results underscore the importance of monitoring VO_2_peak in these individuals [47]. Although the relationship between VO_2_peak and BMI is already known in healthy individuals, it is important to highlight this association in individuals with PCS. This is because the correlation remained significant even when 85% of our sample was composed of overweight or obese individuals. A recently published multicentre, randomised, clinical trial demonstrated that outpatient treatment with metformin reduced the incidence of PCS by 41% [49].

Regarding resting lung function, most individuals in this study did not exhibit spirometric alterations, which is consistent with the findings of Jimeno-Almazán et al. [50] and Suppini et al. [51]. However, more recent studies by Lo et al. [52] and Huang et al. [53] identified spirometric alterations in a significant proportion of individuals. It is important to note that although the sample did not exhibit significant spirometric alterations, these alterations were observed on IOS. In fact, 40% of the participants showed signs of SAD, while only about 16% showed spirometric disturbances. This emphasises the importance of incorporating this technique into clinical practice for patients with PCS [53,54]. Similarly, patients with SAD as measured by IOS had lower VO_2_peak values than those without SAD, suggesting that SAD may indicate worse CPF in this population [55].

It is important to highlight the relationship between Fres—a parameter in IOS indicative of SAD—and VO_2_peak that was observed in our study. Since Fres increases with peripheral airway obstruction due to mucus accumulation, as well as with fibrosis due to the loss of ventilated alveolar units [30], these changes may ultimately result in reduced CPF. Of note, the effect of isolated SAD on exercise is not fully understood. IOS appears sensitive enough to detect changes in Rrs and Xrs during exercise, even in healthy individuals [56]. However, Holley et al. [57] observed that, in a young, active population with exercise limitations and normal spirometry and diffusion capacity of the lungs for carbon monoxide (DLco) at rest, SAD measures using IOS were not independently associated with the ventilatory response to exercise.

As expected for this population, the present study demonstrated B-lines, coalescent B-lines, consolidations, and pleural thickening via LUS. Gil-Rodríguez et al. [58] and Botacconda et al. [59] conducted a systematic review describing the usefulness of LUS in patients after a SARS-CoV-2 infection. This study demonstrated the sensitivity and specificity of the technique. Previous research by our group found that LUS can be used for long-term assessment and has potential prognostic value for stratifying patients with PCS [59].

The present study observed showed a significant negative correlation between LUS and VO_2_peak. Though small, this correlation indicates that the observed changes may contribute to the reduction in CPF. This could be due to impaired ventilation and gas diffusion during exertion in these individuals [60,61]. Additional studies reinforce the role of LUS in assessing individuals with PCS. Giovannetti et al. [62] and Clofent et al. [63] demonstrated that LUS can be used as an initial exam to identify residual lung alterations consistent with CT findings. A systematic review with meta-analysis highlighted the relevance of LUS in investigating persistent post-COVID dyspnea by identifying associated interstitial and pleural patterns [64]. Because LUS is a portable, radiation-free, and easy-to-use instrument, evidence-based protocols and updated international guidelines recommend it as a safe and efficient method for diagnosing and monitoring these individuals [64,65,66,67]. Our study also observed a significant correlation between low ventilatory reserve and the LUS aeration score. This finding reinforces the idea that ventilatory limitation is one possible mechanism of exercise intolerance in patients with PCS.

The strength of this study is that it assessed SAD through the IOS and its correlation with VO_2_peak, the gold standard for measuring the CPF under exertion. However, the limitations of the present study should be noted. First, the study was cross-sectional, making it impossible to establish a cause-and-effect relationship. Second, we did not use a control group for comparison. This is important because people with reduced CPF (even without PCS) are more likely to have structural lung impairment and altered resting lung function [68]. Furthermore, it would be important to have a control group to help interpret our results because changes in CPX and IOS values can occur in healthy individuals due to a loss of efficiency in alveolocapillary oxygen balance during exercise [56,57]. Third, we did not perform blood gas analyses, which could have been useful for determining whether the observed abnormalities resulted from a central phenomenon, such as a loss of efficiency in oxygen uptake, or a peripheral phenomenon, such as muscle fatigue. The latter depends on limiting the diffusion-perfusion of oxygen in the lungs at the alveolar level, as well as the oxygen supply to the muscles [39]. Fourth, DLco and body plethysmography were not measured because, during the pandemic, the laboratory chose to reduce the risk of contamination through aerosol generation. This could have strengthened the correlation with CPF. Fifth, peripheral and respiratory muscle strength were not assessed, both of which are linked to CPF. Finally, no analysis was performed to stratify whether participants had received any vaccine doses during the study, when vaccines became available. Despite these limitations, our findings reinforce the idea that age, BMI, respiratory mechanics, and LUS signs correlate with CPF. Our results provide a basis for further investigation into the mechanisms behind reduced CPF during exertion in patients with PCS. This research could be applied in clinical trials with this patient population and in clinical rehabilitation practice.

## 5. Conclusions

People with PCS who undergo CPX show changes in CPF. In these individuals, altered LUS and IOS may contribute to a lower VO_2_peak. Furthermore, VO_2_peak worsens with age and higher BMI. While our results are promising, more research is needed to determine whether interventions that optimise small airway function can improve CPF in individuals with PCS.

## Figures and Tables

**Table 1:** Anthropometric data, clinical characteristics, comorbidities, practice of physical activity and physical therapy (*n* = 60).

Variable	
Female (*n*, %)	39 (65)
Age (years)	50 ± 12.1
Weight (kg)	81.8 ± 17.4
Height (cm)	165.8 ± 9.9
BMI (kg/m^2^)	29.8 ± 5.3
Etnia birracial (*n*, %)	35 (58.3)
Hypertension (*n*, %)	25 (41.7)
Diabetes (*n*, %)	15 (25)
Physical activities (*n*, %)	35 (58.3)
Hospitalisation related to COVID-19 (*n*, %)	15 (25)
Mechanical ventilation related to COVID-19 (*n*, %)	4 (6.7)
Physical therapy related to COVID-19 (*n*, %)	23 (38.3)

Results expressed as the mean ± standard deviation or number (%). BMI, body mass index.

**Table 2:** Results of the cardiopulmonary exercise test, pulmonary function test at rest and aeration score on the lung ultrasound.

Variable	Values
**Cardiopulmonary exercise test**	
VO_2_peak (mL/kg/min)	18.4 ± 5.9
VEpeak (L/min)	60.7 ± 44.2
Ventilatory reserve (L)	20.5 ± 11
HRpeak (beats/min)	141 ± 23.9
PuO_2_peak (mL/beats)	13 ± 3.5
RR (breaths/min)	37.1 ± 8.94
FeO_2_ (%)	683 ± 79
Peak wordload (Watt)	75.6 ± 25
Total time (min)	7.5 (3.5–12.5)
METs	5.25 ± 1.74
**Spirometry**	
FVC (% predicted)	87.4 ± 8.4
FEV_1_ (% predicted)	87.8 ± 8.2
FEV_1_/FVC (%)	82.5 ± 6.3
FEF_25–75%_ (% predicted)	88.3 (78.7–120.5)
**Impulse oscillometry**	
Fres (Hz)	17.5 (12.4–21.9)
R5 (cmH_2_O/L/s)	4.5 (3.8–6)
R5 (% predicted)	142 (119–163)
R20 (cmH_2_O/L/s)	4.2 (3.4–5.3)
R20 (% predicted)	140 (114–171)
R5–R20 (cmH_2_O/L/s)	0.2 (−0.2–1)
**Aeration score, n (%)**	
Low	57 (95%)
Moderate	1 (1.6%)
Severe	2 (3.3%)

Results expressed as the mean ± standard deviation or as the median and interquartile range. VO_2_, oxygen uptake; VE, minute ventilation; HR, heart rate; PuO_2_: oxygen pulse (VO_2_peak/HRpeak); RR, respiratory rate; FeO_2_, expired O_2_ fraction; METs, metabolic equivalent of task; FVC, forced vital capacity; FEV_1_, forced expiratory volume in one second; FEF_25–75%_, forced expiratory flow during the middle half of the FVC manoeuvre; Fres, resonance frequency; R5, respiratory system resistance at 5 Hz; R20, respiratory system resistance at 20 Hz; R5–R20, heterogeneity of resistance between 5 Hz and 20 Hz.

**Table 3:** Spearman’s correlation coefficients between peak oxygen consumption, clinical characteristics, pulmonary function parameters at rest, and aeration score on lung ultrasound.

Variable	r_s_	Values
Age (years)	−0.465	**0.0002**
BMI (kg/m^2^)	−0.354	**0.006**
**Spirometry**		
FVC (% predicted)	0.212	0.10
FEV_1_ (% predicted)	0.213	0.10
FEV_1_/FVC (%)	0.052	0.69
FEF_25–75%_ (% predicted)	0.131	0.32
**Impulse oscillometry**		
Fres (Hz)	−0.312	**0.019**
R5 (cmH_2_O/L/s)	0.002	0.99
R20 (cmH_2_O/L/s)	−0.012	0.93
R5–R20 (cmH_2_O/L/s)	−0.041	0.76
**Aeration score**	−0.261	**0.044**

BMI, body mass index; FVC, forced vital capacity; FEV_1_, forced expiratory volume in one second; FEF_25–75%_, forced expiratory flow during the middle half of the FVC manoeuvre; Fres, resonance frequency; R5, respiratory system resistance at 5 Hz; R20, respiratory system resistance at 20 Hz; R5–R20, heterogeneity of resistance between 5 Hz and 20 Hz. The significant *p*-value is in bold.

## Data Availability

The original contributions presented in this study are included in the article. Further inquiries can be directed to the corresponding author.

## Abbreviations

The following abbreviations are used in this manuscript:

BMI	Body mass index
CPF	Cardiopulmonary fitness
CPX	Cardiopulmonary exercise test
CT	Computed tomography
DLco	Diffusion capacity of the lungs for carbon monoxide
FEF_25–75%_	Forced expiratory flow during the middle half of the FVC manoeuvre
FeO_2_	Expired O_2_ fraction
FEV_1_	Forced expiratory volume in one second
Fres	Resonance frequency
FVC	Forced vital capacity
HR	Heart rate
IOS	Impulse oscillometry system
LUS	Lung ultrasound
METs	Metabolic equivalent of task
MVV	Maximal voluntary ventilation
PCS	Post-COVID syndrome
R5	Resistance at 5 Hz
PuO_2_	Oxygen pulse
R20	Resistance at 20 Hz
RR	Respiratory rate
Rrs	Resistance of respiratory system
SAD	Small airway dysfunction
VE	Minute ventilation
VO_2_	Oxygen consumption
Xrs	Reactance of respiratory system
